# Low-Temperature Deposition of Transparent Conducting Films Applied to Flexible Electrochromic Devices

**DOI:** 10.3390/ma14174959

**Published:** 2021-08-31

**Authors:** Ke-Ding Li, Po-Wen Chen, Kao-Shuo Chang

**Affiliations:** 1 Department of Materials Science and Engineering, National Cheng Kung University, Tainan 70101, Taiwan; tyty01068@gmail.com (K.-D.L.); kschang@mail.ncku.edu.tw (K.-S.C.); 2 Division of Physics, Institute of Nuclear Energy Research, Taoyuan City 32546, Taiwan

**Keywords:** transparent conducting oxides (TCOs), indium zinc tin oxide (IZTO), electrochromic devices (ECDs)

## Abstract

Here, we compare two different transparent conducting oxides (TCOs), namely indium tin oxide (ITO) and indium zinc tin oxide (IZTO), fabricated as transparent conducting films using processes that require different temperatures. ITO and IZTO films were prepared at 230 °C and at room temperature, respectively, on glass and polyethylene terephthalate (PET) substrates using reactive magnetron sputtering. Electrochromic WO_3_ films deposited on ITO-based and IZTO-based ECDs using vacuum cathodic arc plasma (CAP) were investigated. IZTO-based ECDs have higher optical transmittance modulation, ΔT = 63% [from T_bleaching_ (90.01%) to T_coloration_ (28.51%)], than ITO-based ECDs, ΔT = 59%. ECDs consisted of a working electrochromic electrode (WO_3_/IZTO/PET) and a counter-electrode (Pt mesh) in a 0.2 M LiClO_4_/perchlorate (LiClO_4_/PC) liquid electrolyte solution with an active area of 3 cm × 4 cm a calculated bleaching time t_c_ of 21.01 s and a coloration time t_b_ of 4.7 s with varying potential from −1.3 V (coloration potential, V_c_) to 0.3 V (bleaching potential, V_b_).

## 1. Introduction

The optical properties (transmittance, reflectance, and absorption) of electrochromic devices (ECDs) can be changed using a dc pulsed voltage [1]. Electrochromism is associated with double injection/extraction of positive ions (lithium or proton) and electrons into/out of electrochromic materials [2]. A wide variety of electrochromic materials have been developed, including metal oxides [3,4,5], small organic molecules [6], and conductive polymer thin films [7,8,9]. In recent years, electrochromic devices (ECDs) have attracted tremendous attention due to their potential applications, such as in smart windows [10,11], optical displays [12] and rear-view mirrors [13]. Smart windows based on electrochromic (EC) materials allow easy control of indoor sunlight and solar heat and can be used to effectively reduce the heating or cooling loads of building interiors [2,14]. Flexible electrochromic devices (FECDs) have become an important demand, and hold new possibilities for the application of ECDs, for example, for thermal control of satellites [15], potentially flexible hidden message displays and wearable smart clothes applications [16,17].

In addition, FECDs are very thin and lightweight, which makes their use more flexible than rigid ECDs. The conventional application of FECDs is seriously restricted by fabrication techniques and electrochromic materials such as flexible transparent conductive films and electrochromic (EC) materials with the desired photoelectrical properties and durability [15,17,18], which is a key point developed in FECDs. Traditional deposition techniques used to fabricate FECDs include magnetron sputtering, plasma-enhanced chemical vapor deposition, electrodeposition and lithography. The advantages of FECDs include the potential to reduce production costs using roll-to-roll deposition [19]. In general, FECDs are composed of anodic and cathodic coloring materials in a five-layer structure [20]. A pair of transparent conducting layers sandwich an ionic conduction layer (electrolyte) in contact with an electrochromic (EC) layer and an ion storage (complementary) layer—TCO/EC/IC/CE/TCO, where TCO, IC and CE are the transparent conducting oxide, the ion conducting layer (electrolyte) and the counter electrode, respectively [20,21]. Tungsten oxide (WO_3_) is known as one of the most popular cathodic coloration materials and nickel oxide (NiO) is a typical anodic coloration material that has been intensively investigated. A popular TCO is indium tin oxide (ITO)—the most widely used material as a transparent conducting film for electrochromic devices. However, ITO films exhibit excellent characteristics only in crystalline forms, and have to be deposited at high temperatures at least above 200 °C to be crystallized [22]. Further, its brittleness and high-temperature processing requirements limit its use in flexible devices [23,24]. Low-temperature deposition of amorphous TCOs (α-TCOs) typically occurs via overlaps of empty isotropic ns orbitals of their heavy metal cations in the electronic configuration (*n* − 1)d_10_ns_0_ (*n* ≥ 4) [25], with excellent characteristics that are comparable with that of crystalline TCOs, and thus this is a promising a new trend in optoelectronic devices. α-TCOs have been found to possess good electrical conductivity, high transmittance, high mobility [26,27,28], high thermal/chemical stability, low deposition temperatures and lower roughness. In recent years, IZTOs film have received much attention as new candidates for transparent conducting films. In this study, we compare ITO and IZTO films as transparent electrodes in ECDs using different deposition processing temperatures. FECD performance was studied as a function of the grain size of the WO_3_ particles deposited on the two different TCO electrodes. We applied ITO and IZTO films to FECDs consisting of a working electrode (WO_3_ electrode film deposited on ITO/glass and IZTO/PET) and a counter electrode (Pt mesh) in a 0.2 M LiClO_4_/perchlorate (LiClO_4_/PC) solution.

## 2. Experimental Methods

### 2.1. Methods

**Figure 1:**
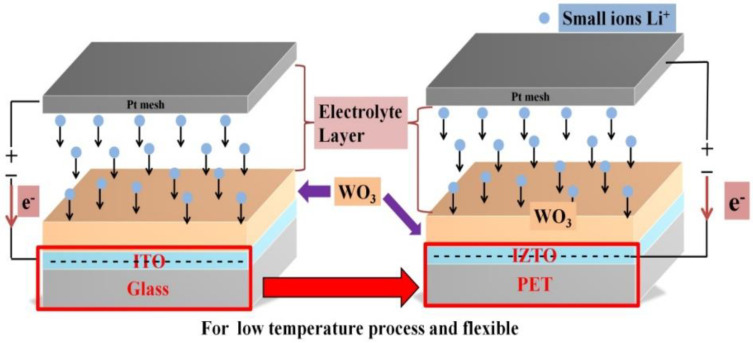
The typical structure of the electrochromic device for coloration states.

The typical structure of Pt mesh/electrolyte layer/EC layer/TCO substrates for coloration state indicates the movement of an injection of positive ions (lithium) and electrons into electrochromic materials as shown in Figure 1. The PET and glass substrates used were thoroughly cleaned beforehand through an ultrasonic bath sequence in acetone, ethanol, and deionized water for 15 min each and were then subsequently dried using high-purity N_2_ gas and loaded into the sputter chamber and the distance between the target and substrate was set at approximately 90 mm. The deposition chamber is then evacuated to a high-vacuum base pressure set to less than 9.33 × 10^−4^ Pa. The working pressure, set at 0.13 Pa, was kept with an argon (Ar) gas flow of 30 sccm. IZTO films were fabricated without substrate heating, while ITO films were fabricated with the substrate heated to 230 °C in the deposition process, and the target was pre-sputtered for 3 min. The individual ITO (90 at.% In_2_O_3_ + 10 at.% SnO_2_) and IZTO (70 at.% In_2_O_3_ + 10 at.% SnO_2_ + 20 at.% ZnO) films were then deposited on the substrates using DC magnetron sputtering of 100 W to achieve a final thickness of approximately 800 nm. The deposition process of TCO films is listed in Table 1. On the finished TCO/glass substrate samples, a WO_3_ electrode film (220 nm) layer was deposited using cathodic arc plasma (CAP) deposition with a high-purity tungsten (W)-metal target (76 mm in diameter and 12 mm in thickness) at room temperature. The base chamber pressure was set at than 1.33 × 10^−3^ Pa using a turbo pump. In this study, we used an oxygen mass flow of 375 sccm and an argon mass flow of 75 sccm for the reactive gases. The WO_3_ films were fabricated on individual TCOs/glass as electrochromic layers, which are listed in Table 2.

### 2.2. Characterization

The crystal structural properties of the TCO films were analyzed by Grazing Incidence X-ray diffraction (GI-XRD). The electrical properties of TCO films were characterized using a Hall effect measurement system at room temperature. We used a UV–vis spectrometer (USB 4000, Ocean Optics, Inc., 830 Douglas Ave. Dunedin, FL, USA) to measure the optical properties of TCO films in the wavelength range of 200–2000 nm. Using Scanning Electron Microscopy (SEM), we observed the surface morphologies of WO_3_ nanoparticles on the different TCO thin films (WO_3_/TCO/PET and WO_3_/TCO/glass). Electrochemical characterization was carried out using chronoamperometry (CA) and electrochemical impedance spectroscopy (EIS) (Autolab, model PGSTAT 30) of WO_3_/TCO/PET or WO_3_/TCO/glass, which were systematically discussed. In the electrolyte system, we used a liquid electrolyte composed of lithium perchlorate (LiClO_4_, Mw = 106.39, Sigma-Aldrich, Darmstadt, Germany) and propylene carbonate (PC, C_4_H_6_O_3_, Sigma-Aldrich), and the resulting weight ratio was 0.053 (LiClO_4_/PC = 10.6 g/200 mL). An active area of 2 × 3 cm^2^ was used in our case for ECDs. The Alpha-Step D-500 stylus profiler is measured with each layer of thickness. In situ optical transmittance was carried out with a U–vis spectrometer. Electrochemical impedance spectroscopy (EIS) of all ECDs was measured with the frequency range set from 1 mHz to 1 MHz.

## 3. Results and Discussion

**Figure 2:**
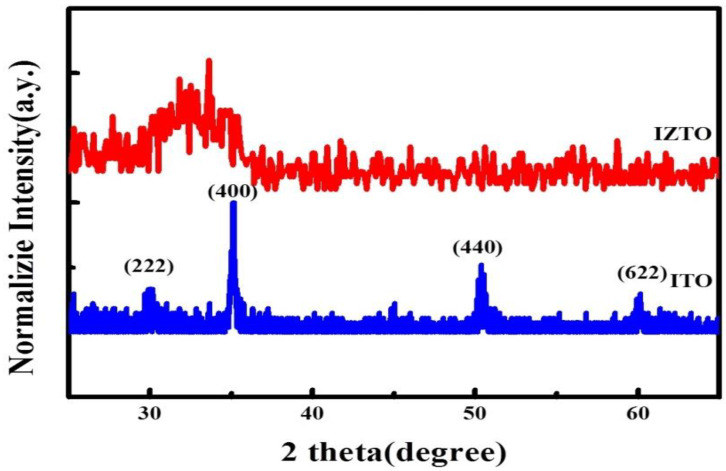
The XRD patterns of the different TCO films.

Figure 2 displays the GI-XRD patterns of ITO/glass and IZTO/PET using DC magnetron sputtering power at 100 W. The GI-XRD patterns of ITO film deposited on glass at 230 °C which exhibited polycrystalline indexed at (211), (222), (400), (440), and (622). The high-intensity peak at 2θ of 35° indicates the crystalline nature of film with (222) as the preferred plane orientation. The GI-XRD patterns of ITZO film deposited on PET substrates at room temperature and exhibited a lone, broad peak at 2θ of 33°, indicating an amorphous structure.

**Figure 3:**
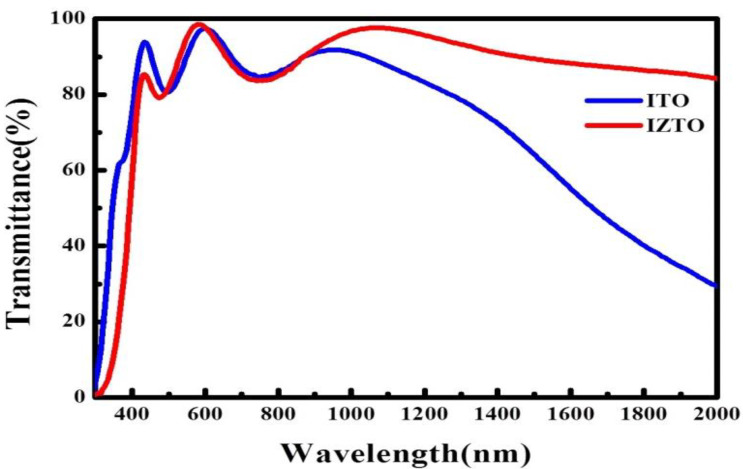
The optical transmittance spectra of the different TCO films.

Figure 3 exhibits the optical transmittance of ITO/glass and IZTO/PET in the wavelength range of 300–2000 nm measured with UV–vis spectrometry. The samples of ITO/glass and IZTO/PET were measured with average transmittance, sheet resistance (Ω/□), electrical resistivity(ρ), mobility (μ), carrier concentration (n) and figure of merit (*Φ_TC_)*. The average transmittance of ITO/glass and IZTO/PET was calculated to be approximately 86.8% and 88.4% in the wavelength range of 400–800 nm. The mobility of IZTO was higher than ITO due to poor electron–phonon scattering because of the amorphous structures. The carrier concentration of IZTO film was lower than the ITO film due to carrier compensation. According to Haacke’s relation, in order to qualify the performance of TCO [24], figure-of-merit *Φ_TC_* can be explored by Equation (1):

*Φ_TC_* = *T*(λ)^10^/R_s_, (1)

where the *T*(λ) is the optical transmittance, R_s_ is the sheet resistance, and *Φ_TC_* is displayed in Table 3; the higher value of *Φ_TC_* is 40 × 10^−3^ for the ITO thin films fabricated with substrate heating of 230 °C than 31.1 × 10^−3^ for the IZTO films fabricated at room temperature.

**Figure 4:**
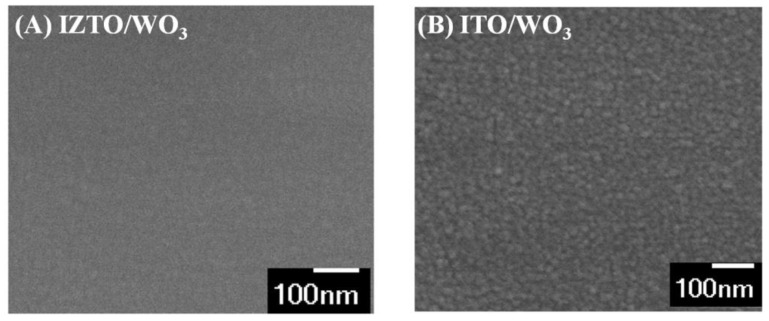
**(A)** SEM images of WO_3_/IZTO/glass and (**B**) SEM images of WO_3_/ITO films.

Figure 4 shows the SEM images of WO_3_ films deposited on ITO/glass and IZTO/PET. From SEM images, we find that the WO_3_ particle of the IZTO-based ECD is smaller than the ITO-based ECD. Patrick et al. [29] suggested that the rough surface raises the local supersaturation in the solution, thus leading to another mechanism of enhanced nucleation rate. The IZTO film was featureless and smooth without defects such as pinholes, cracks, and protrusion [30] that could control the WO_3_ particle size in furthering the process.

We sought to elucidate the electrochemical properties of the WO_3_/ITO/glass and WO_3_/IZTO/PET by constructing three electrode cells, which comprised a working electrode (WO_3_ film on TCO substrate), a counter-electrode (Pt mesh) and a reference electrode (Ag/AgCl) in a 0.2 M LiClO_4_/PC solution [31,32].

**Figure 5:**
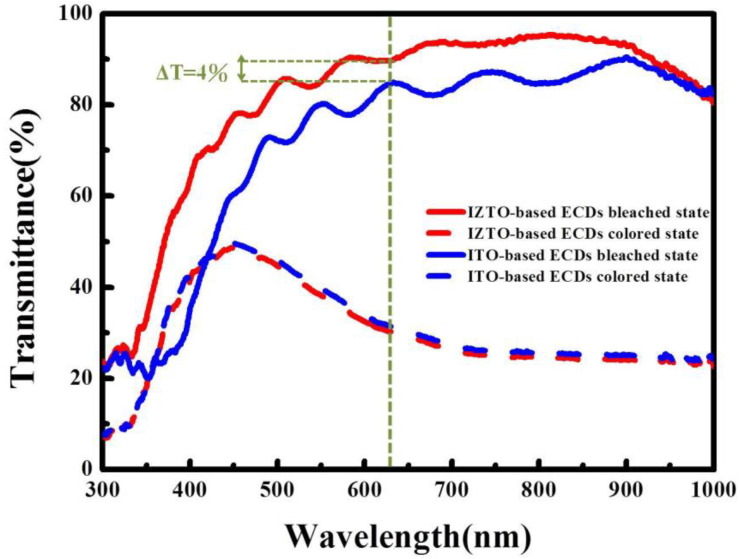
The transmittance of IZTO-based and ITO-based ECDs for bleaching and coloration states.

Figure 5 displays the CA curves at a voltage of 0.3 V (bleaching state) to −1.3 V (coloring coloration state) with a pulsed time interval of 30 s for the coloration and bleaching states of the optical transmittance spectra of WO_3_/ITO/glass and WO_3_/IZTO/PET in the range from 300 to 1000 nm. Optical transmittance modulation (∆T = T_bleaching_ − T_coloration_) of IZTO-based ECD and ITO-based ECD varied at 63% and 59%. The transmittance optical modulation, ∆T = 63% between coloration and bleaching states, with IZTO-based ECDs higher than ITO-based ECDs at a fixed wavelength of 633 nm due to relevant surface electrochromic materials (WO_3_/ITO/glass and WO3/IZTO/PET) for particle size discussed in detail below.

FECDs involved the double injection/extraction of positive ions (lithium or proton) and electrons into/out of the host WO_3_ lattice in the transition from W^5+^ to W^6+^. This follows the electrochemical reaction:

WO_3_ (bleaching state) + x (Li^+^ + e^−^) → Li_x_WO_3_ (coloration state), (2)

during applying the negative voltage, the reduction of W ions W^6+^ to W^5+^ led to the coloration state, and the reverse voltage the oxidation of W^5+^ to W^6+^ to the bleaching state.

**Figure 6:**
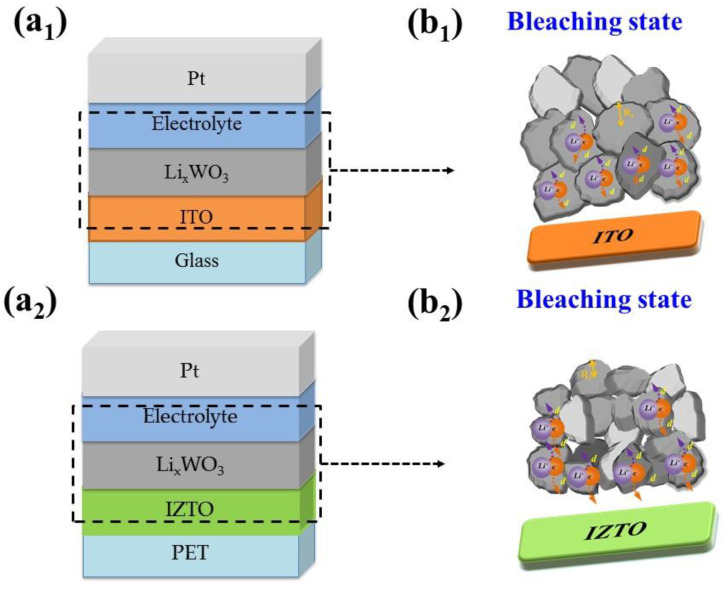
Schematic diagram of electron and Li^+^ ions transport in the ECDs. The schematics of Li ions path through surface morphology with different grain type (**a_1_**) ITO based ECDs (**a_2_**) IZTO based ECDs. (**b_1_**,**b_2_**) Li+ ions and electrons can extract a distance of d out Li_x_WO_3_, and R_1_ and R_2_ rep-resent the radius of Li_x_WO_3_ particle for ITO-based and IZTO-based ECDs.

To clarify the outstanding optical transmittance difference of IZTO-based ECD and ITO-based ECD, the behaviors of Li^+^ ions and electrons in the transport process were considered. Figure 6 shows the bleaching state for electron and Li^+^ ions extraction transport in electrochromic WO_3_ films when applying the positive voltage. Li^+^ ions and electrons can extract a distance of d out Li_x_WO_3_ (where d represents the maximum electron and Li^+^ ions from the interior of Li_x_WO_3_, R_1_ and R_2_ represent the radius of Li_x_WO_3_ particle for ITO-based and IZTO-based ECDs, respectively.) It presumes R_2_ (the radius of Li_x_WO_3_ particle for IZTO-based ECDs) is smaller than d (the distance of Li^+^ ions and electrons can extract), which can explain electron and Li^+^ ions can be extracted from the interior of Li_x_WO_3_ for IZTO-based ECDs. However, R_1_ (the radius of Li_x_WO_3_ particle for ITO-based ECDs) is larger than d (the distance of Li^+^ ions and electrons can extract), and this means electron and Li^+^ ions cannot be extracted to the electrolyte from the Li_x_WO_3_ particle completely, indicating the Li_x_WO_3_ particles have some residual W^5+^ ions in the process of fading oxidation that W^5+^ cannot completely convert to W^6+^, leading to poor optical transmittance modulation [31,32].

**Figure 7:**
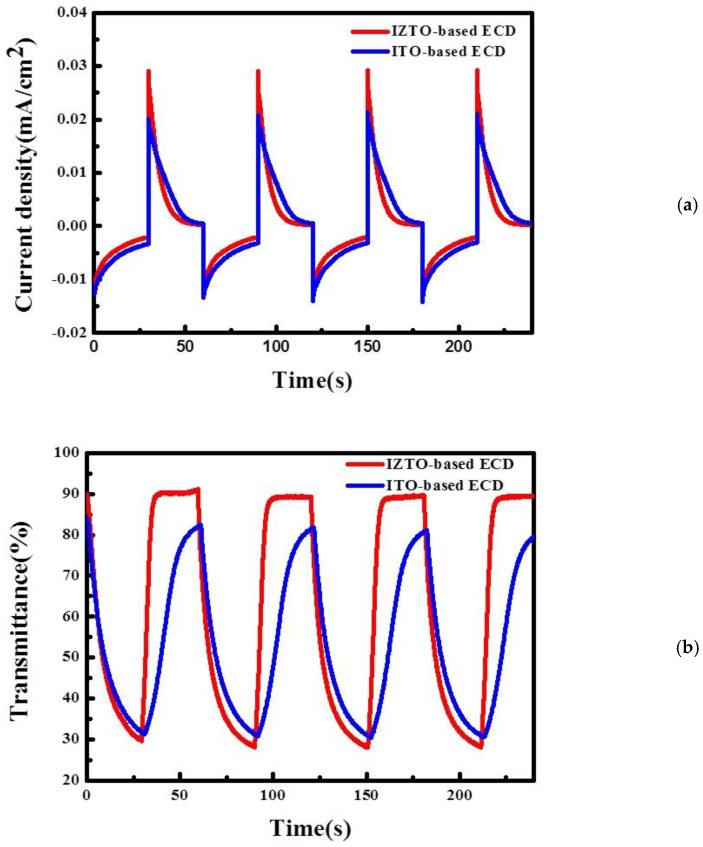
(**a**) The current density; (**b**) the variation in the optical transmittance at 633 nm for IZTO-based and ITO-based ECDs.

Figure 7 shows CA curves with varying potential from −1.3 V to 0.3 V for 30 s and the corresponding in situ transmittance response of IZTO-based ECD and ITO-based ECD at a fixed wavelength of 633 nm. Furthermore, the switching response times of the coloration and bleaching states are defined as reaching 90% of the full transmittance modulation investigated in detail in Table 4 [33]. In this study, IZTO-based ECD and ITO-based ECDs were calculated as a t_c_ of 21.01 s and 20.13 s, and as t_b_ of 4.7 s and 22.32 s, respectively. In general, the bleached response time presented faster coloration kinetics than coloration due to the lower conductivities of the bleached-state forms than those of the colored-state forms [33]. The bleaching time is determined by the dispersion coefficient of ions and the length of dispersing channels [34,35]. The fast bleaching time of IZTO-based ECDs is associated with the WO_3_ particle size due to the shorter dispersing channels.

**Figure 8:**
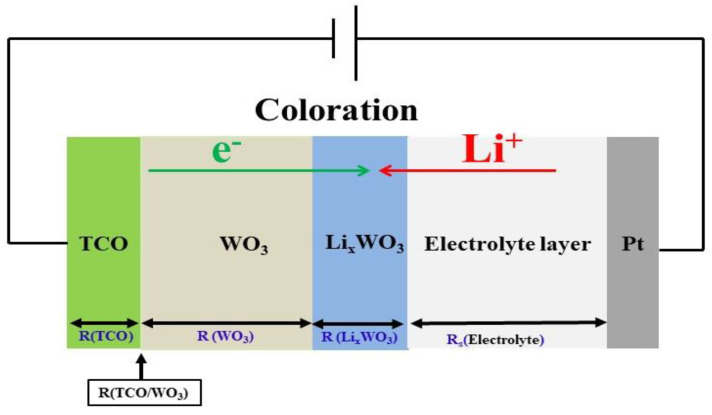
A schematic of the electrochemical reaction for WO_3_ films in an electrolyte which is conductive by applying potential.

Figure 8 shows a schematic of the electrochemical reaction for the Pt mesh/electrolyte layer/EC layer/TCO substrate at the coloration state, which can be expressed as the sum of the following [36]

R = R_s_ (electrolyte) + R_ct_ (Li_x_WO_3_) + R (WO_3_) + R (TCO/WO_3_) + R (TCO) (3)

The ions for the intercalation process are supplied using an Electro-Chemical Impedance spectroscopy (EIS) tool to analyze the ion charge transfer properties of IZTO-based and ITO-based ECDs. Further, we compare the charge transport kinetics of the two TCO electrodes based on ECDs via a Nyquist curve analysis.

**Figure 9:**
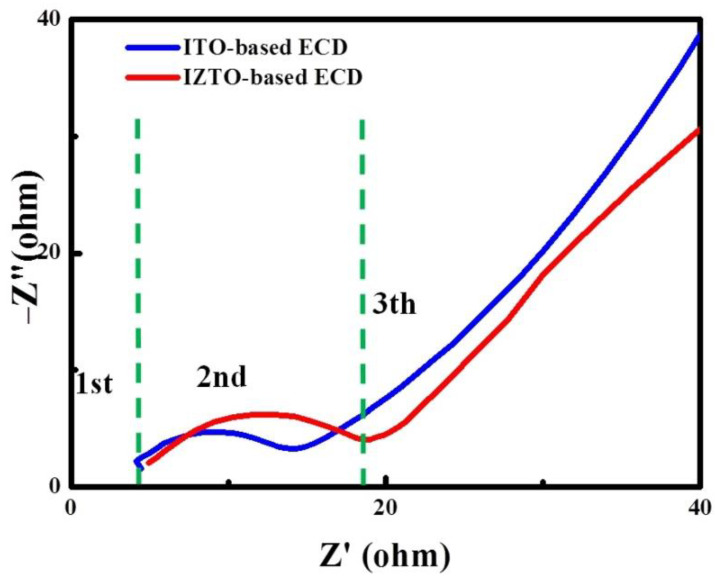
Nyquist plots of IZTO-based and ITO-based ECDs.

Figure 9 shows that there are three processes involved in the intercalation of ions into electrochromic WO_3_ films. First, the left point of the semi-circle, in the high-frequency region, indicates the migration of ions at the electrode–electrolyte interface. Secondly, the middle of the semicircle, in the middle-frequency region, corresponds to the charge-transfer process. Third, the right part of the semi-circle, in the low-frequency region, indicates the migration of ions at the electrode–electrolyte interface. Electrolyte solution resistance R_s_ was found in the high-frequency region in intercept of the semi-circle real impedance axis on the Nyquist curve [37]. The R_s_ value of IZTO-based ECD was approximately 4.9 Ω, which is similar to the ITO-based ECD (4.5 Ω). The semicircle in the middle frequency region can be ascribed to the charge-transfer interphase resistance R_ct_ of the Li^+^ ions dependent on the diameter of the semicircle across the interphase electrolyte and WO_3_ films. The results show that the ITO-based ECD reveals a larger semi-circle with approximately 9.5 Ω resistance that was smaller than the IZTO-based ECD of 13.5 Ω, and is demonstrating a smaller charge carrier interphase resistance. The inclined line indicates the diffusion of the Li^+^ ions into the WO_3_, associated with Warburg impedance, Z_w_ corresponding to the inclined straight line in the low-frequency region in the Nyquist plot [37,38,39,40]. The fast coloration time of ITO-based ECD is associated with sheer resistance and charge-transfer interphase resistance due to the smaller Li^+^ ions and electron diffusion barrier.

The coloration efficiency (CE) is a very important electrochromic property of ECDs and it is associated with the optical density (ΔOD) and the intercalated charges (*Q_in_*) [39]. It can be obtained by Equation (4).

CE = ΔOD/*Q_in_* (4)

**Figure 10:**
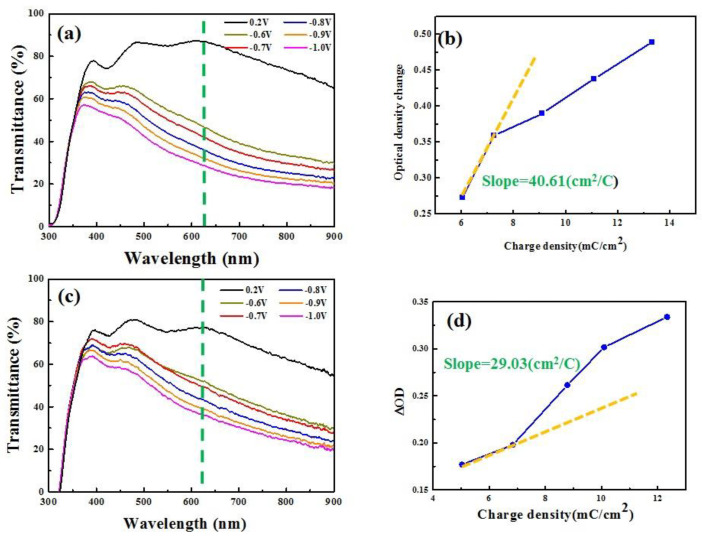
(**a**,**c**) The transmittance spectra of IZTO-based and ITO-based ECDs in the range from 300 to 900 nm using varying voltage from 0.2 V to −1.0 V. (**b**,**d**) ΔOD versus charge density at 633 nm of IZTO−based and ITO−based ECDs.

ΔOD = log_10_ (T_bleached_/T_colored_) (5)

where the ΔOD is the optical density variation between the transmittance values in the bleached and colored states at 633 nm. The amount of charge intercalated (*Q_in_*) when a negative potential is applied is integrated by CA measurement. A high CE value (cm^2^ C^−1^) is achieved when a large change in optical density is driven by a low amount of inserted charge. Figure 10a,c show the coloration and bleaching states of the optical transmittance spectra of WO_3_/IZTO/PET and WO_3_/ITO/glass in the range from 300 to 900 nm using varying voltage from 0.2 V (bleaching state) to −1.0 V (coloration state) with a pulsed time interval of 60 s. Figure b,d show the dependence of ΔOD on intercalated charges density at a wavelength of 633 nm. The calculated CE result for the IZTO-based ECD is 40.61 cm^2^ C^−1^, higher than ITO-based ECD (29.03 cm^2^ C^−1^) because of the smaller WO_3_ particle size to provide larger optical transmittance modulation.

**Figure 11:**
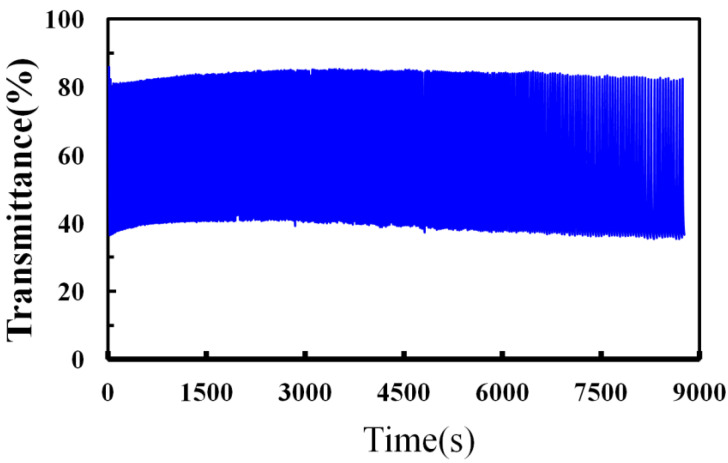
Life cycle measurements for the IZTO-based ECDs by CA measurements under 0.2 V~−1.0 V for 30 s.

The cyclic durability of IZTO-based ECDs was also studied by a CA, measured applying 0.2 V to −1.0 V with a pulsed time interval of 30 s. The transmittance spectra in Figure 11 show the 450 coloration–bleaching cycles, which vary as a function of time within 9000 s for the IZTO-based ECD. As shown in Figure 11, the IZTO-based ECD has a stable electrochromic performance from the first cycle to 450 cycles.

## 4. Conclusions

In conclusion, electrochromic WO_3_ films were deposited by CAP on ITO/glass prepared by heating the substrate and the IZTO/PET fabricated at room temperature. First, WO_3_/IZTO/PET shows a smaller grain size on the amorphous IZTO compared with crystal ITO for WO_3_/ITO/glass and was found to present wider optical modulation in the visible light region (~63% at 633 nm) and higher coloration efficiency (40.61 cm^2^ C^−1^) than ITO-based ECDs. Second, amorphous IZTO is a suitable alternative to other TCOs prepared using higher-temperature processes. CE for IZTO-based ECD is 40.61 cm^2^ C^−1^, higher than ITO-based ECD (29.03 cm^2^ C^−1^) because of the smaller WO_3_ particle sizes to provide larger optical transmittance modulation. IZTO-based ECDs have stable electrochromic performance from the first cycle to 450 cycles.

## Figures and Tables

**Table 1:** Details of the parameters of IZTO and ITO films.

Processing	Working Pres. (Pa)	Base Pres. (Pa)	Ar (sccm)	DC Power (W)	Thickness (nm)	Deposition Temp. (°C)
IZTO	0.13	9.33 × 10^−4^	30	100	800	RT
ITO	0.13	9.33 × 10^−4^	30	100	800	230

**Table 2:** Deposition parameters of the WO_3_ films.

Target	Working Pres. (Pa)	Base Pres. (Pa)	Ar/O_2_ (sccm)	Power (W)	Thickness (nm)	Deposition Temp. (°C)	Deposition Rate (nm/min)
Metal W	2.7	1.3 × 10^−3^	0.2	1350	220	RT	14.67

**Table 3:** The comparison of Hall measurement results, transmittance, and Φ_TC_ values of the different TCO films.

Processing	Transmittance (%)	Sheet Resistance (Ω/□)	Electrical Resistivity (Ω·cm)	Mobility (cm^2^·V^−1^)	Carrier Concentration (cm^−3^)	Figure of Merit (Ω^−1^)
IZTO	86.8	7.8	6.2 × 10^−4^	6.622	8.40 × 10^20^	31.1 × 10^−3^
ITO	88.4	7.2	5.7 × 10^−4^	2.95	2.08 × 10^21^	40 × 10^−3^

**Table 4:** The switching response times of the coloration and bleaching states for IZTO-based and ITO-based ECDs.

Samples	Bleaching Time, t_b_ (s)	Coloration Time, t_c_ (s)
ITO based ECDs	22.32	20.13
IZTO based ECDs	4.7	21.01

## Data Availability

The data presented in this study are available on request from the corresponding author. The data are not publicly available due to privacy.
